# A Case Report of Takotsubo Cardiomyopathy Masquerading as Acute Coronary Syndrome: A Diagnostic Challenge

**DOI:** 10.7759/cureus.96238

**Published:** 2025-11-06

**Authors:** Pavankumar Narayanan, Mohammed M Ali, Vinod Warrier, Nayeem M Aoyon, Alekya Siddabathula, Saravanaa Sankar

**Affiliations:** 1 Department of Acute Medicine, Southend University Hospital, Mid and South Essex NHS Foundation Trust, Southend-on-Sea, GBR; 2 Department of Emergency Medicine, Mercy Hospital St. Louis, St. Louis, USA

**Keywords:** acute coronary syndrome, apical akinesia, down-trending troponins, echocardiography, stress-induced cardiomyopathy, takotsubo cardiomyopathy

## Abstract

Presentations of Takotsubo cardiomyopathy (TTC) may resemble those of acute coronary syndrome (ACS), including symptoms such as severe chest pain, elevated troponins, and dynamic ECG changes. Early recognition is crucial to avoid unnecessary interventions.

We report the case of a 70-year-old female who presented with sudden-onset severe left-sided chest pain, markedly elevated but down-trending troponins, and an ECG showing dynamic, diffuse T-wave inversions. Echocardiography revealed preserved basal contraction with apical akinesia and an ejection fraction (EF) of 25%. Coronary angiography demonstrated unobstructed coronaries. She was initially managed medically under the ACS protocol with careful risk stratification until the diagnosis was confirmed on coronary angiography, after which she was managed conservatively. Follow-up echocardiography at 4 weeks showed recovery of EF to 55% without intervention, confirming TTC.

ACS-like presentations with down-trending troponins, basal contraction with apical akinesia on echocardiography, diffuse ECG changes, and resolving chest pain may represent TTC. Careful risk stratification can guide the safe timing of angiography, avoid unnecessary urgent transfer to the nearest percutaneous coronary intervention (PCI) centre, and ensure continued medical ACS management until the diagnosis is confirmed on coronary angiography combined with left ventriculography.

## Introduction

Takotsubo cardiomyopathy (TTC), also known as stress-induced cardiomyopathy or “broken heart syndrome,” is a transient form of acute left ventricular dysfunction driven by catecholamine excess that results in direct myocardial stunning; nevertheless, the precise association remains incompletely understood [1]. This has been demonstrated on imaging studies such as iodine-123 metaiodobenzylguanidine (¹²³I-MIBG) myocardial scintigraphy, which show cardiac sympathetic hyperactivity despite preserved coronary perfusion, supporting a neurogenic contribution to myocardial stunning. Epicardial and microvascular coronary spasm, reduced coronary flow reserve, and sympathetically mediated microcirculatory dysfunction have all been suggested as contributory mechanisms [1]. This condition predominantly affects postmenopausal women, possibly due to reduced estrogen-mediated cardioprotection combined with increased catecholamine sensitivity and autonomic dysfunction following physical or emotional stress [2]. Although typically reversible, its initial presentation closely mimics acute coronary syndrome (ACS), with chest pain that is heavy, squeezing, or crushing, typically central or epigastric, and may radiate to the arms [1], along with dynamic ECG changes and elevated cardiac biomarkers [3,4]. Other life-threatening complications include arrhythmias, QT prolongation, and torsades de pointes [1].

TTC is increasingly recognized in clinical practice and is estimated to account for approximately 2% of all patients presenting with suspected ACS [5]. This overlap presents a diagnostic dilemma in the emergency setting, as immediate decisions about antiplatelet therapy, anticoagulation, and the need for urgent percutaneous coronary intervention (PCI) must be made before the diagnosis is clarified [6].

While coronary angiography remains essential to exclude obstructive coronary artery disease and diagnose TTC, additional features including down-trending troponins, non-territorial wall-motion abnormalities on echocardiography, and exposure to physical or emotional stress may raise suspicion for TTC [7]. The 2023 European Society of Cardiology (ESC) guidelines for ACS recommend that patients without very high-risk features undergo invasive angiography within 24-72 hours, allowing time for careful risk stratification in ambiguous cases [8].

We present the case of a 70-year-old woman who developed sudden, severe, left-sided chest pain with markedly elevated troponins, diffuse T-wave inversions, and severe left ventricular systolic dysfunction but was ultimately diagnosed with TTC. This case highlights the importance of considering TTC in ACS-like presentations, where thoughtful risk stratification can help avoid unnecessary emergency PCI while ensuring patient safety.

## Case presentation

This is a 70-year-old female with a past medical history of Ménière’s syndrome and asthma, and a significant family history of premature ischemic heart disease in her father before the age of 60. She was physically active at baseline and fully independent, with no restrictions in daily activities.

The patient presented with sudden-onset, severe, left-sided chest pain, described as pressure-like and rated 10/10 in intensity, which began while showering shortly after mild exercise in a swimming pool followed by sauna use. The pain was non-radiating and persisted for three hours, showing only partial relief to 8/10 despite administration of morphine, aspirin, nitrates, and oxygen therapy, before gradually subsiding to 1/10 over the next 12 hours. She also reported a milder episode of similar chest discomfort five days earlier. Notably, the chest pain was not accompanied by diaphoresis, dyspnoea, palpitations, nausea, vomiting, or radiation to the arms, jaw, or back.

**Figure 1:**
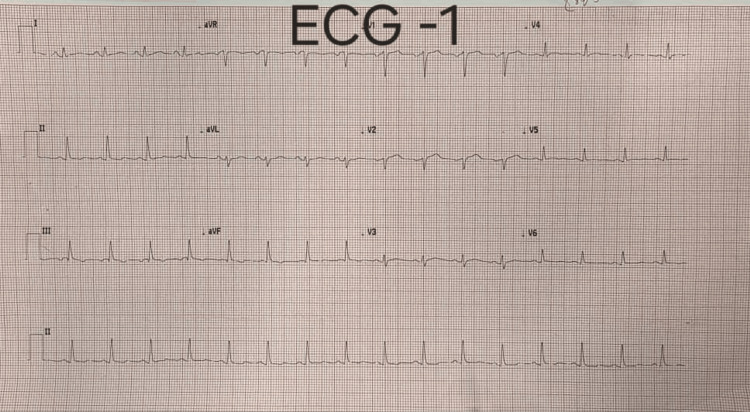
T-wave flattening observed in leads V4-V6, III, and aVF. aVF: Augmented voltage foot lead.

**Figure 2:**
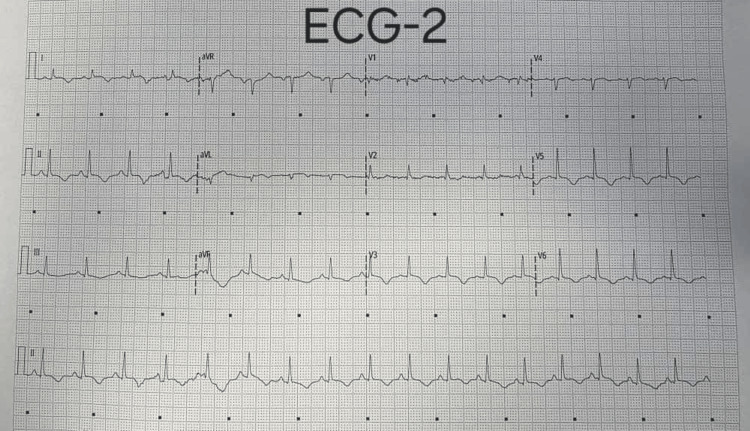
Serial ECG demonstrating dynamic T-wave inversions in leads V3-V6, I, II, III, aVL, and aVF, with a positive T wave in aVR. aVL: Augmented voltage left arm lead; aVF: Augmented voltage foot lead; aVR: Augmented voltage right arm lead.

On admission, the ECG demonstrated dynamic and diffuse T-wave inversions affecting all leads except V1-V2 (Figures 1-2).

On examination, the patient was alert, oriented to time and place, and hemodynamically stable initially, with a blood pressure of 117/89 mmHg, which subsequently declined to 84/47 mmHg, indicating potential left ventricular failure. Cardiovascular, respiratory, and systemic examinations were unremarkable, with no murmurs, additional heart sounds, or signs of heart failure. A chest X-ray demonstrated clear lung fields bilaterally, with no evidence of pulmonary congestion or consolidation. Laboratory investigations revealed a markedly elevated high-sensitivity troponin T level of 1536 ng/L, which down-trended to 515 ng/L over the next 12 hours, alongside normal CRP and renal function tests (Table 1). Given the acute chest pain and transient hypotension, pulmonary embolism (PE) was considered a differential diagnosis; therefore, D-dimer testing was performed and found to be normal (53 ng/mL), helping to exclude PE. Transthoracic echocardiography demonstrated concentric left ventricular remodeling with preserved basal contraction but akinesia involving the apical and mid-left ventricular segments, resulting in severe left ventricular systolic dysfunction with an estimated ejection fraction of approximately 25% (Video 1).

**Table 1:** Key laboratory investigations on admission. hs-Troponin T: High-sensitivity troponin T; D-dimer: Fibrin degradation product; ALT: Alanine aminotransferase; HDL: High-density lipoprotein; LDL: Low-density lipoprotein.

Description	Result	Normal values
1st hs-Troponin T	1536 ng/L	<14 ng/L
n	515 ng/L	<14 ng/L
D-dimer	53 ng/mL	<243 ng/mL
CRP	2 mg/L	<5 mg/L
Total bilirubin	46 µmol/L	0-21 µmol/L
ALT	15 U/L	<35 U/L
Alkaline phosphatase	65 U/L	30-130 U/L
Sodium	133 mmol/L	133-146 mmol/L
Potassium	4.3 mmol/L	3.5-5.3 mmol/L
Urea	7.5 mmol/L	2.5-7.8 mmol/L
Creatinine	75 µmol/L	45-83 µmol/L
Cholesterol	3.5 mmol/L	<5.0 mmol/L
HDL	1.70 mmol/L	≥1.2 mmol/L
LDL	1.35 mmol/L	<3.0 mmol/L
Triglycerides	1.06 mmol/L	<2.26 mmol/L

**Video 1:** Transthoracic echocardiography showing preserved basal contraction with apical and mid-ventricular akinesia, and severely reduced left ventricular systolic function (ejection fraction = 25%). TTC: Takotsubo cardiomyopathy.

The patient was immediately managed as a non-ST-elevation myocardial infarction (NSTEMI), receiving a loading dose of aspirin 300 mg orally, ticagrelor 180 mg orally, fondaparinux 2.5 mg subcutaneously, atorvastatin 80 mg, and a beta-blocker orally for rate control, given her elevated heart rate. The nearest cardiothoracic centre was contacted, and the patient’s clinical status was reviewed in detail. In light of the settling chest pain, down-trending troponin levels, stabilizing T-wave inversions on ECG, and echocardiographic findings, urgent ambulance transfer for immediate PCI was deferred, as the case was considered a completed myocardial infarction with no anticipated benefit from emergency revascularization.

**Figure 3:**
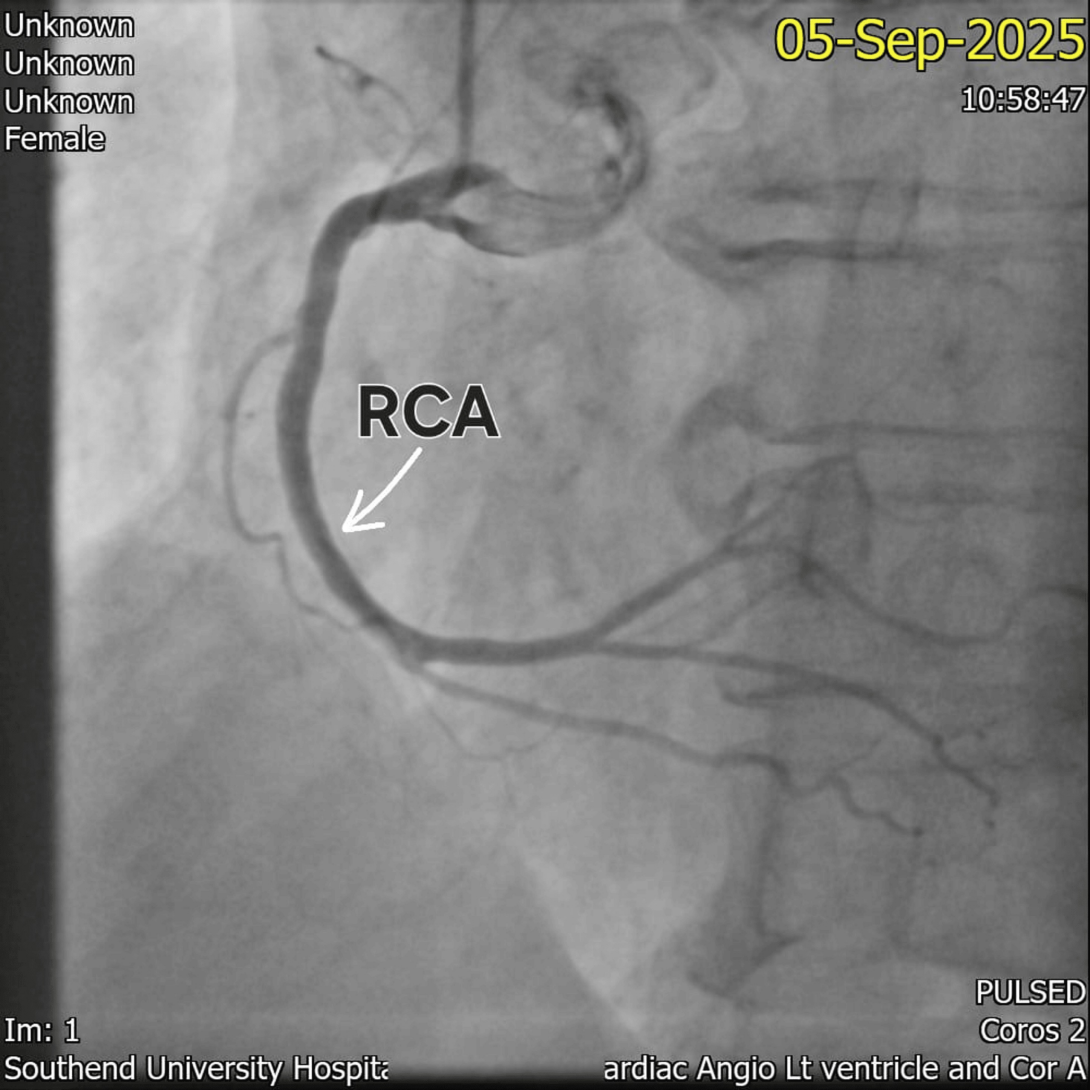
Coronary angiography demonstrating an unobstructed right coronary artery (RCA).

**Figure 4:**
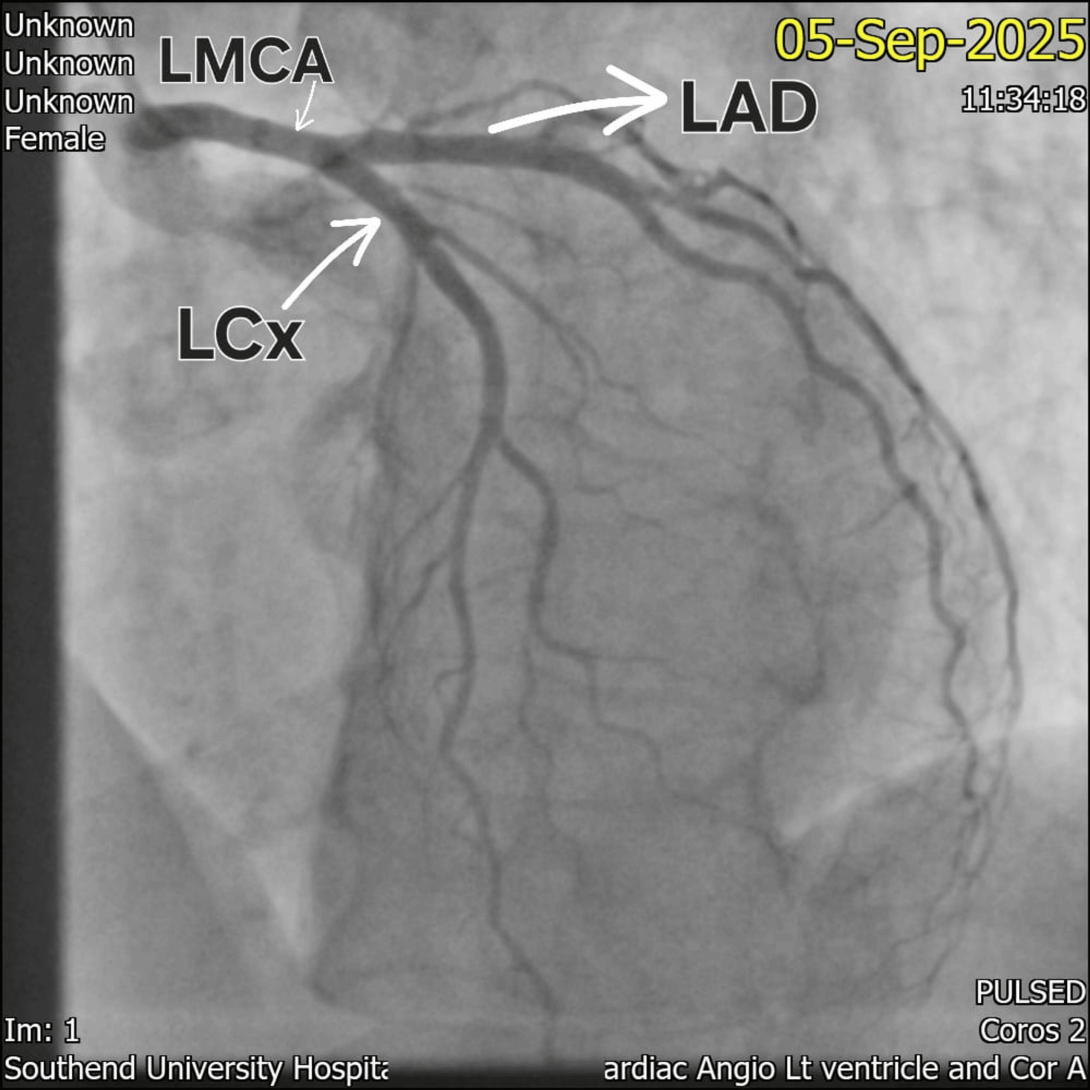
Coronary angiography demonstrating unobstructed left anterior descending (LAD) and left circumflex (LCx) arteries. LMCA: Left Main Coronary Artery.

Coronary angiography performed the following day (Figures 3-4) revealed unobstructed left and right coronary arteries. Left ventriculography, however, was not carried out due to the occurrence of catheter-induced spasm during the procedure, limiting direct visualization of the apical ballooning typically associated with TTC.

The patient was discharged on a low-dose ACE inhibitor and scheduled for repeat echocardiography at four weeks, which demonstrated improvement in ejection fraction to 55% (Video 2), confirming reversible myocardial dysfunction. Cardiac MRI was not performed, as the patient had claustrophobia. During detailed history-taking, the patient reported experiencing significant financial stress in recent weeks, which likely served as the emotional trigger, in addition to possible physical exertion, for her TTC, highlighting the role of psychosocial factors in precipitating stress-induced cardiac events. Although a formal psychiatric consultation was not undertaken during the current admission, she was advised to seek mental health support through her primary care provider should symptoms of anxiety or emotional distress persist.

**Video 2:** Transthoracic echocardiography at 4-week follow-up showing normal left ventricular size with concentric remodeling and improved systolic function (ejection fraction 50-55%).

## Discussion

TTC can closely mimic ACS, as patients often present with sudden-onset chest pain, markedly elevated troponin levels, and dynamic electrocardiographic changes. This overlap with ACS presents a significant diagnostic challenge in the acute setting, as immediate management decisions regarding antiplatelet therapy, anticoagulation, and percutaneous coronary intervention (PCI) are often required before the diagnosis is clarified [2-3].

Echocardiography provides critical diagnostic information, typically showing preserved basal contraction with akinesia or hypokinesia of the apical and mid-left ventricular segments, often not confined to a single coronary territory [3-4]. This non-territorial pattern contrasts with the regional wall-motion abnormalities seen in myocardial infarction secondary to coronary occlusion. Coronary angiography further aids in differentiation, as patients with TTC usually have unobstructed coronary vessels [3-4]. In our patient, angiography confirmed the absence of obstructive coronary disease, and left ventriculography was not performed due to catheter-induced spasm, although echocardiographic findings were highly suggestive of typical TTC.

Risk stratification plays a pivotal role in guiding management decisions, particularly regarding the timing of invasive angiography. Tools such as the Global Registry of Acute Coronary Events (GRACE) score [8-10], combined with hemodynamic assessment and evaluation for ongoing ischemia, including persistent chest pain, dynamic ECG changes, and rising troponins, allow clinicians to identify patients at intermediate or high risk who may safely defer emergent PCI [8-9]. In this case, the patient’s GRACE score ranged from 138 to 149, depending on blood-pressure measurements, indicating intermediate-to-high risk [6]. Careful application of these criteria permitted deferral of urgent transfer for intervention, which is reserved for the very high-risk category of patients, while continuing standard ACS management medically, followed by invasive coronary angiography within 24-72 hours.

The natural course of TTC is typically reversible, with left ventricular ejection fraction (LVEF) generally improving on follow-up echocardiography within four to eight weeks [10]. In our patient, LVEF improved from 25% to 55% at four-week follow-up without any coronary intervention, confirming the diagnosis of TTC. Emotional and physical stressors, such as the significant financial strain reported by this patient, are common precipitants and underscore the importance of detailed psychosocial assessment in suspected cases [2-4].

Clinically, follow-up imaging, particularly echocardiography, is critical to document recovery and guide ongoing management, including heart-failure therapy if required [11]. Patients should also be safety-netted regarding symptoms of heart failure and advised to seek urgent medical attention if they develop worsening dyspnoea, orthopnoea, peripheral edema, syncope, or recurrent chest pain suggestive of reduced ejection fraction or clinical deterioration.

## Conclusions

This case highlights that ACS-like presentations with down-trending troponins, echocardiography showing preserved basal contraction with apical akinesia (non-region-specific regional wall motion abnormality), and ECG demonstrating diffuse T-wave inversions may represent TTC. Careful risk stratification based on the GRACE score, hemodynamic status, and evidence of ongoing ischemia, assessed through persistent chest pain, dynamic ECG changes, or rising troponins, can guide the safe timing of invasive angiography while ensuring continued ACS management until the diagnosis is confirmed. The improvement in ejection fraction from 25% to 55% over four weeks without intervention confirms the reversible nature of stress-induced cardiomyopathy.
